# Overlapping and distinct phenotypic profiles in Alzheimer’s disease and late onset epilepsy: a biologically-based approach

**DOI:** 10.3389/fneur.2023.1260523

**Published:** 2024-03-13

**Authors:** Anli A. Liu, William B. Barr

**Affiliations:** ^1^Langone Medical Center, New York University, New York, NY, United States; ^2^Department of Neurology, School of Medicine, New York University, New York, NY, United States; ^3^Neuroscience Institute, Langone Medical Center, New York University, New York, NY, United States

**Keywords:** late onset epilepsy, Alzheimer’s disease, neuropsychology, mild cognitive impairment, memory, language, hippocampus, interictal epileptiform discharge

## Abstract

Due to shared hippocampal dysfunction, patients with Alzheimer’s dementia and late-onset epilepsy (LOE) report memory decline. Multiple studies have described the epidemiological, pathological, neurophysiological, and behavioral overlap between Alzheimer’s Disease and LOE, implying a bi-directional relationship. We describe the neurobiological decline occurring at different spatial in AD and LOE patients, which may explain why their phenotypes overlap and differ. We provide suggestions for clinical recognition of dual presentation and novel approaches for behavioral testing that reflect an “inside-out,” or biologically-based approach to testing memory. New memory and language assessments could detect—and treat—memory impairment in AD and LOE at an earlier, actionable stage.

## 1 Introduction

Both patients with Alzheimer’s disease and epilepsy patients report difficulty with episodic memory, or remembering autobiographical events (see Box 1) (1, 2). Common complaints including forgetting conversations, losing personal items, or repeating questions or stories. Besides similarity in clinical presentation, the epidemiological, pathological, and neurophysiological overlap between Alzheimer’s Disease and late-onset epilepsy (LOE, first seizure after the age of 60) has been well-described (3–5) (see Box 1). Epidemiologically, AD patients have a seizure incidence of 12%–28% (6), while patients with LOE have a 3-fold higher risk of developing dementia (3). After a diagnosis of LOE, patients have a median time of 3.66 years to dementia ascertainment (2).

When should clinicians suspect AD pathology in the older patient presenting with their first lifetime seizure, and seizures in dementia patients? The authors propose that understanding the shared and distinct biology between AD and LOE will improve diagnosis and management, especially during early stages of each condition.

BOX 1 **Definitions**

**Episodic Memory** is memory for personally-experienced events, for example, what one ate for lunch, a conversation with a friend, a movie narrative, and or the birth of one’s child. These memories may include people, context, perceptual detail, timing and sequence, emotion, and meaning. Episodic memory includes 3 phases: encoding, consolidation, and retrieval. The hippocampus is thought to be critically involved in these three stages during waking and sleep. Patients with hippocampal dysfunction, such as patients with Alzheimer’s Disease, traumatic brain injury, and temporal lobe epilepsy commonly report memory impairment as a cognitive comorbidity.

**Alzheimer’s Disease** (AD) is the most common neurodegenerative disorder in the US, affecting 1 in 9 people aged 65 or older in the US (6.7 million). Age is the greatest risk factor for AD: 13.1% of people ages 75 to 84, and 33.3% of people age 85 or older have AD (1). Classically, AD presents as impairment in episodic memory function, then language and executive function. While there are no effective cures, there are medications which can slow cognitive decline or address comorbid symptoms. Recent work suggests that between 12% and 28% of patients with AD have seizures arising from the mesial temporal lobe (7).

**Early onset Alzheimer’s Disease (EOAD)** is the clinical presentation of Alzheimer’s Disease before the age of 65. Clinical features and pathology are similar to late-onset Alzheimer’s disease (LOAD).

**Amyloid Beta**. Accumulation of the protein beta-amyloid outside neurons defines early pathophysiological changes in AD. Extracellular a-beta accumulation is associated with neuronal cell dysfunction, inflammation, and cell death (8).

**Tau**. Tau is an intracellular microtubule associated protein whose pathologic accumulation impairs intracellular function, including glucose transport, and contributes to direct neural degeneration. Phosphorylated tau (p-tau) is seen multiple degenerative and epilepsy conditions, including AD, movement disorders, temporal lobe epilepsy, post-traumatic epilepsy, autism, Dravet’s syndrome, focal cortical dysplasia, and tuberous sclerosis (9).

**Seizures** are events of abnormal sustained electrical activity in the brain that manifest silently or as sudden changes in awareness, sensation, movement, or behavior. Seizures can be provoked by transient medical factors such as excessive alcohol, recreational drug use, or infection. Seizures can also recurrent event arising from abnormal brain activity.

**Epilepsy** is a neurological disease defined by the potential for recurrent seizures, and may be treated by medications, devices, or surgery. Epilepsy can be caused through many mechanisms, such as genetics, developmental malformations, traumatic brain injury, or stroke. A Many patients with epilepsy do not have a known cause to seizures, and are classified as “cryptogenic-onset epilepsy”.

**Late-Onset Epilepsy (LOE)** is presentation of first seizure in an epilepsy patient after the age of 60. In two thirds of cases of LOE, a structural cause can be determined, such as cerebrovascular disease (stroke, 30%–50%), neurodegenerative disease (10%–20%), traumatic brain injury (TBI, ≤25%) and brain tumors (10%–30%). Other less common causes of seizures are infection, drug and alcohol toxicity and withdrawal, and autoimmune encephalitis (10).

**Late-Onset Epilepsy of Unknown Etiology (LOUE)**. For the one-third of patients without an identified structural cause, or cryptogenic epilepsy, occult cerebrovascular disease and/or prodromal neurodegenerative disease, are highly suspected.

In this review, we will describe the pathological, neurophysiological, and neuroimaging overlap between AD and LOE. With this biological foundation, we review their cognitive phenotypes as revealed in neuropsychological testing and suggest a few diagnostic approaches. Finally, we propose new behavioral assays that reflect an “inside-out,” or biologically-based approach to testing memory. New memory assessments could be used to detect—and treat—memory impairment in AD and LOE at an earlier, actionable stage (11–13).

Search terms used in PubMed for this review include Alzheimer’s disease; late-onset epilepsy; early-onset epilepsy; Alzheimer’s Disease pathology; late-onset epilepsy; early onset-Alzheimer’s; late-onset epilepsy clinical presentation; neuropsychology and epilepsy; neuropsychology and Alzheimer’s disease; cognitive and epilepsy; cognitive and Alzheimer’s disease; cognitive phenotype and epilepsy; cognitive phenotype and Alzheimer’s disease; memory and epilepsy; memory and Alzheimer’s disease; naming and epilepsy; naming and Alzheimer’s disease; language and epilepsy; language and Alzheimer’s disease; executive functions and epilepsy; executive functions and Alzheimer’s disease; Natural Language Processing and Epilepsy; Natural Language Processing and Alzheimer’s Disease; Automated Speech Analysis and Alzheimer’s Disease; and Eye tracking and Memory.

The authors acknowledge the heterogeneity in AD presentation and etiology but will focus this review on typical AD, which presents with memory dysfunction as a chief complaint. AD includes both early and late onset AD, which share pathology and clinical features. Atypical presentations of AD, or “non-amnestic” AD have been estimated to comprise less than one-third of young AD patients (<65 years) (14), and thus only 6%–7% of the total AD cohort. While atypical AD is a rare but important condition to recognize, we will focus on typical AD and its overlap with LOE. Likewise, familial (i.e., genetic etiology due to APP, PSEN1, or PSEN2 mutations) and sporadic AD share similar neuropathology and clinical features, but familial AD presents earlier. Because familial AD is relatively rare (5% of total AD prevalence), we will not treat familial AD separately from sporadic AD (1).

Furthermore, the terminology LOE will be used in this paper to include both known and unknown causes of seizures in older age. AD pathology can co-exist with other known structural causes in older age, especially vascular etiologies such as stroke or microvascular disease. AD pathology may also comprise a meaningful portion of the one-third of older patients with epilepsy of unknown cause. Of note, TLE is the dominant cohort of focal epilepsy patients and represents the largest cohort of LOE cases (15). The grouping of late-onset temporal lobe epilepsy (TLE) and TLE has been used in neuroimaging and neuropsychology, and thus will be used in this review.

## 2 Shared pathological processes in AD and LOE

AD and LOE mainly affect temporal lobe and specifically hippocampus (5, 15, 16) at early stages. Several MRI, pathological, neurophysiological, and behavioral studies demonstrate the pathophysiological overlap between the AD and LOE pathways, which may explain similar cognitive presentations (17, 18).

### 2.1 Pathological amyloid and tau accumulation

Similar accumulation patterns of extracellular amyloid-beta peptides (A-beta) and intracellular tau tangles (Box 1) (8, 19) that have been well described in the AD population with recent rodent and human work suggesting a similar process in LOE patients (19). Amyloid precursor protein (APP) is an essential membrane glycoprotein that supports numerous physiological functions, including neuronal development, signaling, and intracellular transport (20). Normally, APP cleavage results in several types of a-beta peptides. An imbalance between a-beta production and degradation and clearance leads to extracellular accumulation in hippocampus, neocortex, and the cerebral vasculature, likely initiating AD (20–22). a-beta accumulation outside of neurons blocks cell to cell signaling in the brain and triggers microglial activation. Chronic low-level inflammation characterizes AD, can overwhelm the glial response, and leads to brain atrophy. A-beta’s role in contributing to hyperexcitability and seizures has recently been reported (8, 13). Pathologically high CSF a-beta levels are measured in 37.5% of LOE patients compared to healthy age-matched controls and are associated with a 3.4-fold higher risk of progression to dementia (23).

**Figure 1:**
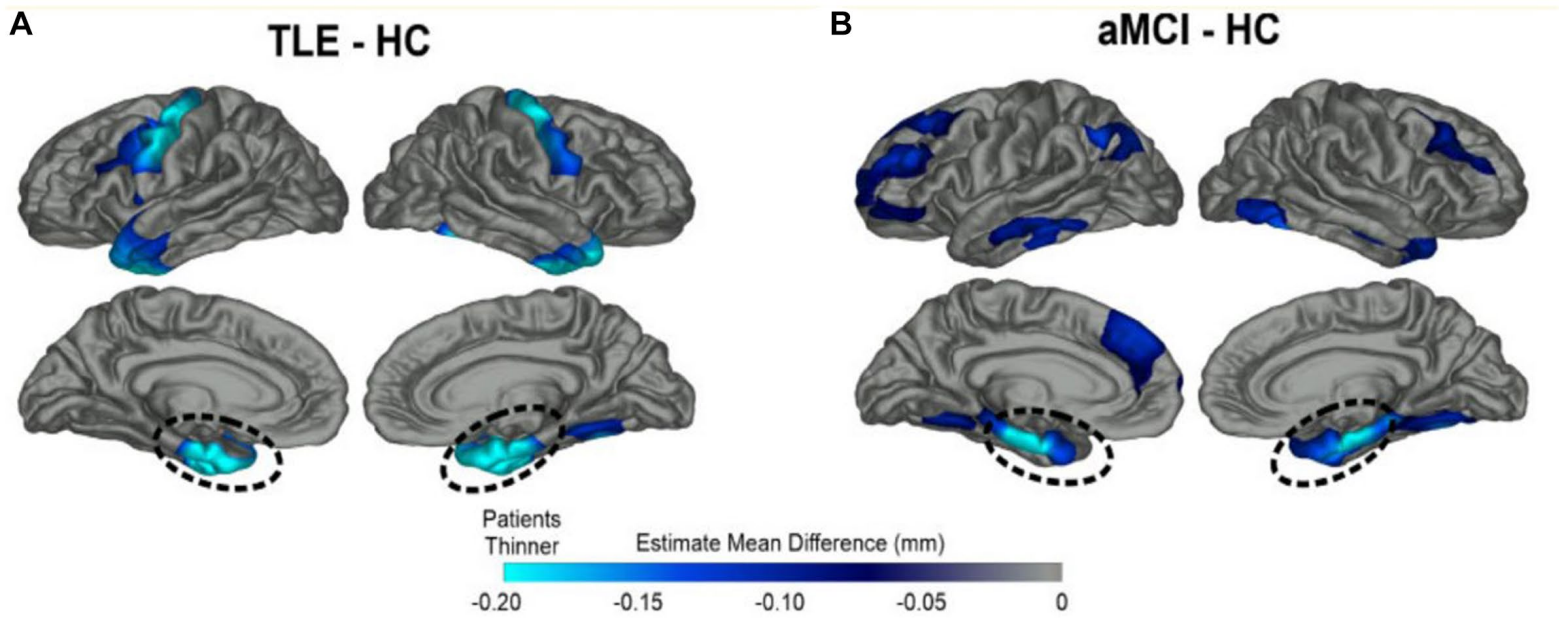
Overlapping patterns of MTL cortical atrophy in TLE and amnestic MCI. Patterns of cortical thinning for **(A)** TLE and **(B)** amnestic MCI patients relative to healthy elderly control subjects (HC). Dark blue represents cortex thinner than healthy controls while turquoise regions demonstrate the most thinning. Both patient groups showed prominent cortical thinning in bilateral medial temporal lobe (MTL) regions highlighted by dashed lines; TLE patients also showed thinning of the primary motor cortex compared to HCs. From Kaestner (2020) with permission (18).

Tau is an intracellular micro-tubule associated protein whose pathologic accumulation results in impairment of intracellular function including glucose transport and direct neural degeneration. Phosphorylated tau (p-tau) is seen multiple degenerative and epilepsy conditions, including AD, movement disorders, temporal lobe epilepsy, post-traumatic epilepsy, autism, Dravet’s syndrome, focal cortical dysplasia, and tuberous sclerosis (9). Examination of resected temporal lobe tissue in a cohort of older TLE patients (*n* = 33, age 50–65) revealed excess tau pathology in 94% of samples (24). Tau burden correlated with the degree of cognitive impairment (24) (Figure 1).

### 2.2 Brain atrophy

Both amnestic mild cognitive impairment (aMCI), the precursor to AD, and LOE patients show atrophy of the bilateral medial temporal lobe structures—including entorhinal, parahippocampal, hippocampal, temporal pole, and fusiform regions (18, 25). LOE patients posssess greater left entorhinal and temporal pole thinning, while patients with amnestic MCI show greater thinning of the bilateral middle temporal cortex and right inferior temporal cortex (Figure 1). Patients with LOE show thinner motor cortex compared to healthy controls (HCs) and amnestic MCI subjects. There has been recent interest in the piriform cortex, a small region sitting adjacent to MTL that supports olfaction and memory and contributes to seizure kindling (26, 27). The piriform cortex is bilaterally atrophied in patients in MCI and AD, and unilaterally atrophied on the side of mesial temporal sclerosis in epilepsy (26).

What is the effect of epilepsy duration? Lifetime seizure frequency may not be the sole driver of cortical thinning (28), as pathological decline can start earlier than clinical presentation in LO-TLE patients (2, 18). Slightly different patterns emerge in early and late onset epilepsy. Patients with LOE demonstrate greater atrophy of the fusiform gyri and similar cognitive profiles compared to patients with early-onset TLE (EO-TLE), even though the latter group endured over 30 years of seizures. As would be expected from this shared pattern of brain atrophy, both LOE and aMCI patients demonstrate memory impairment compared to HCs (17, 18).

### 2.3 Patterns of hippocampal subfield dysfunction

**Figure 2:**
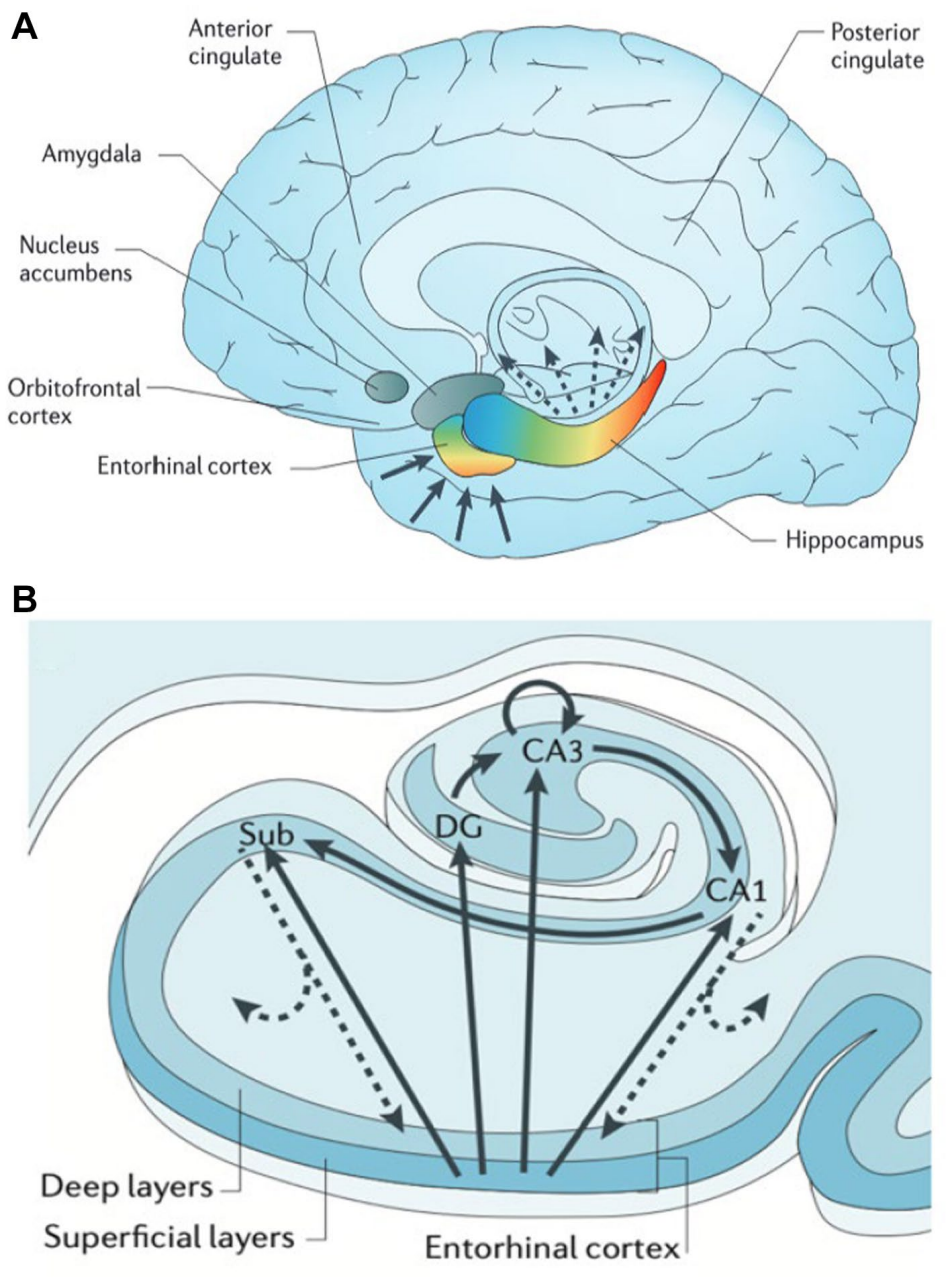
Hippocampal functional organization. **(A)** The hippocampus receives and delivers input from cortex in a topographically organized manner. Anterior cortical regions such as amygdala and frontal lobe relay information directly and indirectly (via entorhinal cortex, EC) to anterior hippocampus (head) and amygdala. Likewise posterior regions such as occipital cortex connect directly and indirectly to posterior hippocampus (tail). **(B)** The hippocampal transverse axis shows how input received by EC is processed within the hippocampus, then delivered back to cortex directly via CA1/subiculum and indirectly through EC with a preserved topographical gradient. From Small (2013) with permission (29).

The hippocampus and adjacent medial temporal lobe structures are affected in AD and temporal lobe epilepsy, as well as several cognitive and psychiatric disorders such as vascular disease, schizophrenia, depression, and PTSD (29). Our understanding of the heterogeneity of cell types, gene expression profiles, and related function has been studied across the long and transverse axes within the hippocampus. The entorhinal cortex (EC) is considered a gateway to the hippocampus, receiving monosynaptic input from various cortical regions, including the perirhinal cortex (the “what” pathway), the parahippocampal cortex (the “where” pathway), the amygdala, and the sensory cortex (Figure 2A). The EC relays this topographically organized information to the hippocampus. Anterior structures such as the amygdala have direct and indirect (via EC) connections to the anterior hippocampus, or head. Conversely, posterior structures such as visual and association cortex have extensive direct and indirect connections with the posterior hippocampus, or tail (29). Along the transverse axis, the entorhinal cortex connects with the dentate gyrus, CA3, CA1, and the subiculum. In the tri-synaptic pathway, information from EC is delivered to DG → CA3 → CA1→ subiculum (Figure 2B). Within CA3, there are auto-association fibers with extensive connections along the hippocampal long axis. Information largely flows out through CA1 and subiculum to be delivered directly or indirectly to cortext through EC, in a topographically preserved manner (29).

**Figure 3:**
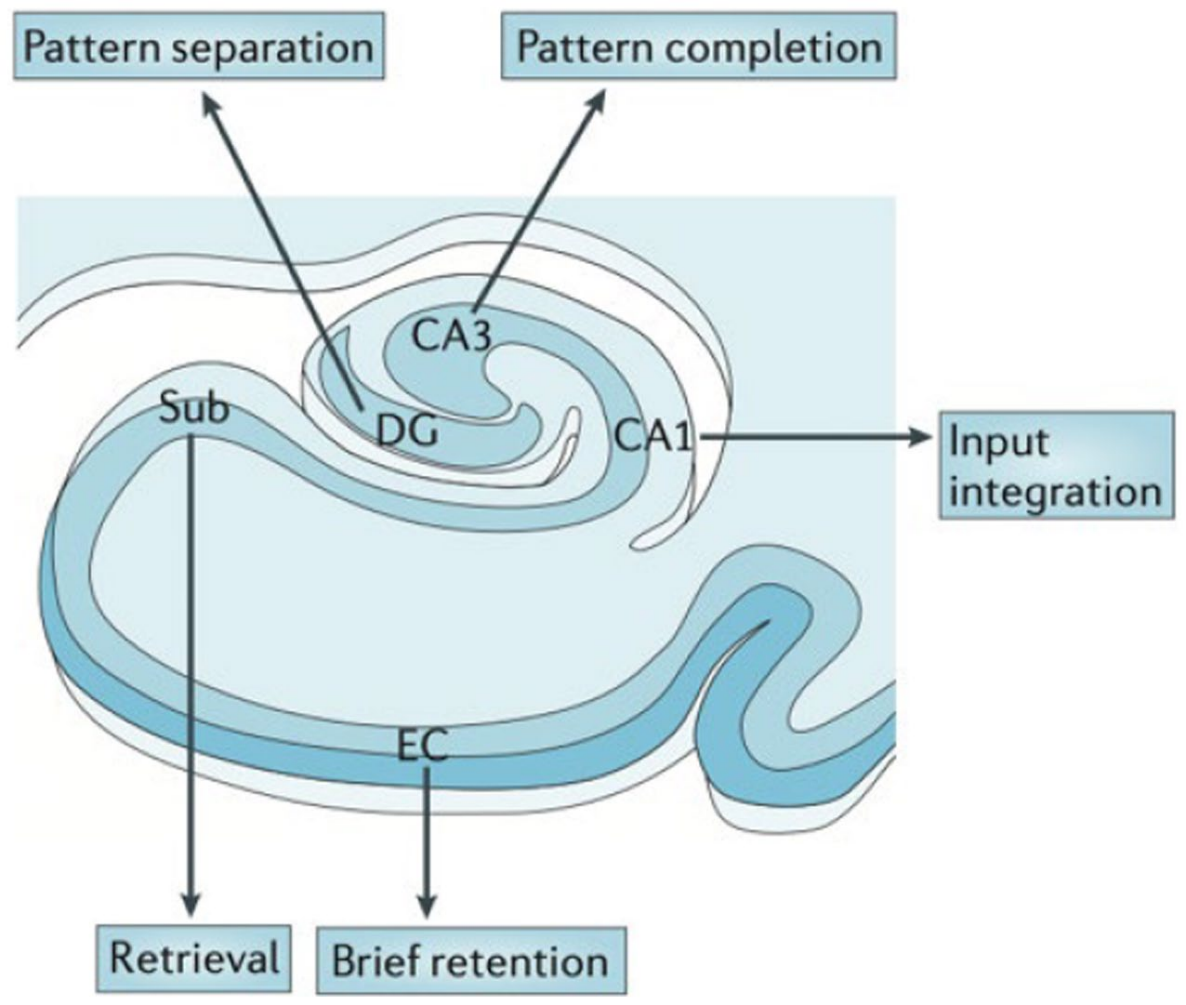
Hypothesized functional organization of the hippocampal transverse axis. Rodent and human studies suggest a functional specialization between the hippocampal subfields. From Small (2023) with permission (29).

High-resolution structural and functional MRI, CT perfusion, and post-mortem studies suggest that hippocampal subfields along the anterior and posterior hippocampus are differentially vulnerable in the spectrum of neuropsychiatric disorders, likely due to differential gene expression profiles (29). A functional differentiation of hippocampal subfields has been proposed (Figure 3), which may be useful to distinguish patient groups. Because each subfield serves as a conduit of information flow, upstream injury will impair downstream functioning and result in more severe memory deficits.

For example, imaging and pathology studies show that the dentate gyrus (DG) is particularly important in “pattern separation,” or representation of similar events as distinct and non-overlapping items (Figure 3) shown in rodents and humans (30–34). DG is particularly vulnerable to aging across species (35). Behaviorally, aged rats and humans have difficulty in distinguishing between similar contexts (36).

**Figure 4:**
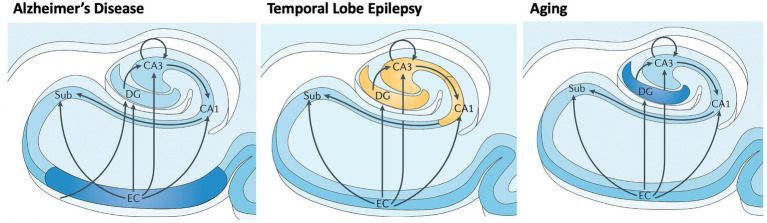
Differential vulnerability of hippocampal subfields in early AD, TLE, and normal aging. Subfields include EC: entorhinal cortex; Sub: subiculurm; DG: dentate gyrus; CA3; CA1. Early involvement of subfields varies between neurological disorders and normal aging, causing local and downstream cognitive dysfunction. Adapted from Small et al. (2013) (29).

AD involves early cell loss in entorhinal cortex (EC) which affects downstream structures such as DG, CA3, CA1, and subiculum (Figure 4), the primary outflow tracts (25, 37). AD patients therefore present with difficulty in all stages of memory, including maintaining information over brief delays (e.g., delayed match to sample tasks, pattern separation deficits, consolidation, and retrieval). In contrast, temporal lobe epilepsy begins with cell loss in dentate gyrus and CA3/CA1, with relatively preserved subiculum, CA2 and EC entorhinal cortex (Figure 4) (38). Therefore, one may expect that TLE patients have difficulty with separation of details (DG), association and consolidation between present and past (CA3/CA1), but less difficulty with forming and retrieving memories *per se*. Both pathological patterns differ from the decline of dentate gyrus function seen in normal aging (35, 39).

Indeed, patients with amnestic MCI have demonstrated poorer delayed memory performance relative to late onset TLE (18, 40). Knowledge of differing subfield patterns of early stages of AD and TLE could be used to design more behaviorally specific tasks to aid in diagnosis and to provide a benchmark for performance (29). Of course, as each of these disease progresses, pathology spreads to nearby regions.

### 2.4 Interictal epileptiform discharges

Interictal epileptiform discharges (IEDs) are pathological bursts of neuronal activity suggestive of cortical hyperexcitability. These subclinical epileptiform events have been observed in 20–50% of AD patients (5, 16, 41), and are associated with accelerated cognitive decline (16). A-beta’s role in contributing to hyperexcitability and seizures has been reported (8, 13). Converging evidence demonstrates that IEDs impair encoding, maintenance, consolidation, and retrieval of verbal material (42–47). Left temporal and parietal neocortical IEDs are associated with impaired memory for word list items and word pairs (44, 47). IEDs outside the seizure onset zone (SOZ) in higher order visual processing regions have been associated with impaired encoding and retrieval performance for words (47). Hippocampal IEDs during encoding of a face-profession pair can reduce odds of recall by 15%; IEDs during recall can reduce odds of recall by 25%, potentially by acutely decreasing hippocampal sharp wave ripples (SWRs). Hippocampal IEDs during sleep impair long-term memory consolidation of verbal and visual material (48). We hypothesize that hippocampal IEDs, prevalent in AD and LOE, can occur during critical memory stages during wake and sleep states to directly compete with physiological processes (49). These interactions contribute to dynamic fluctuations in memory function, and a potential target for closed loop neurostimulation protocols to remediate memory function.

## 3 Characterizing AD and LOE cognitive phenotypes

While neuroimaging and neurophysiology (i.e., EEG) play an essential role in making the clinical diagnosis of AD or LOE, neuropsychological assessment is the gold standard for the assessment, characterization, and tracking of cognitive impairment.

The impairments arising in these conditions differ in the onset, severity, and course from the decline seen in normal aging, including decreased processing speed, memory, language, and executive function (50). Episodic memory and language are the neurocognitive domains most affected in typical AD and TLE and emphasized during neuropsychological testing in both clinical and research settings. Episodic memory has been evaluated by using list-learning, story recall, and figural reproduction tasks (e.g., Rey Auditory Verbal Learning Test & Wechsler Memory Scale) (51). Language tasks have included measures of picture naming (e.g., Boston Naming Test) and verbal fluency (e.g., letter & category). Measures of executive functioning, including tests of rapid mental tracking and problem solving (e.g., Trail Making Test & Wisconsin Cart Sorting Test) are also used in patients with other etiologies such as frontotemporal or vascular dementia. The neuropsychological tests used today for these purposes are criticized for using outdated methodology and for extensive time required to administer and score the tests (52).

Patients with AD and TLE both exhibit deficits on neuropsychological tests requiring recall of newly learned material after a delay period of 20-min or more (53, 54). Results from neuropsychological studies show subtle but important differences in the cognitive presentation between these two groups. Deficits in episodic memory, or difficulty remembering personally experienced events, are commonly the first manifestation of AD. This deficit may involve a combination of reduced encoding of new information and a disturbance of the ability to transfer that information into long-term storage, or a consolidation deficit (54). These cognitive deficits may be due to early involvement of entorhinal cortex (hippocampal input and output, short term retention), (Table 1; Figures 3,4).

**Table 1:** Patterns of cognitive impairment seen in Alzheimer's disease and temporal lobe epilepsy using traditional neuropsychological testing.

Neuropsychological domain	Representative neuropsychological tests	Alzheimer's disease	Temporal lobe epilepsy
Episodic memory	List learning (e.g., RAVLT)	Reduced encoding of new information and consolidation information into long term storage (55).	Intact encoding; Disruption in consolidation of newly learned information (56). Material specific impairments in verbal and visual memory in lateralized cases (57).
	Story recall (e.g., WMS LM)		
	Figural reproduction (e.g., RCFT)		
Language	Picture naming (e.g., BNT)	Deficits in confrontation naming and verbal fluency secondary to a loss of semantic knowledge stores (55).	Intact knowledge stores with a primary difficulty in retrieving lexical and semantic information (61).
	Letter fluency (e.g., COWAT)		
	Category fluency (e.g., Animal naming)		
Executive functions	Trail making test (TMT)	Mild deficits that do not extend to the severity observed in cases of frontotemporal dementia FTD (62).	Mild deficits that do not extend to the severity observed in cases of frontal lobe epilepsy (63).
	Sorting tests (e.g., WCST)		
	Planning tests (e.g., TOL)		

The memory deficits seen in TLE are believed to result from difficulty consolidating newly learned information. On testing, this is displayed as rapid forgetting (58). These could be secondary to early involvement of dentate gyrus/CA3 and CA1, subfields responsible for pattern separation and integration (Figures 3,4; Table 1) (29, 35). Furthermore, difficulty with long term consolidation could be secondary to increased frequency of interictal discharges during NREM sleep (59, 60) or decreased spindle activity seen during NREM sleep (61). Additionally, TLE patients with unilateral onset of left or right hemisphere seizures may exhibit a material-specific impairment in memory for verbal or nonverbal material (62). Finally, there is converging evidence suggesting that subclinical discharges may disrupt consolidation processes causing accelerated rates of forgetting in both conditions (48, 63).

Patients with AD are believed to progress to more widespread and profound declines in language and other domains as the neuropathology spreads from medial temporal lobe structures to association cortices of the temporal, frontal, and parietal lobes (55). Deficits in confrontation naming and semantic fluency (i.e., number of words generated within a category such as animals or fruits) result from loss of semantic knowledge stores. In contrast, naming deficits present when seizures arise from the language dominant hemisphere and are characterized by a deficit in semantic retrieval (64). Executive dysfunction can be identified in early and later stages of both conditions, but is milder than the executive dysfunction associated with other variants of dementia and epilepsy, including frontotemporal dementia (FTD) and frontal lobe epilepsy (FLE) (65, 66).

The profiles of neurocognitive disturbance in AD and TLE are generally studied at the group level. Individual patients exhibit a more heterogenous profile of deficits in episodic memory, language, and executive function than what is reported in group studies. Neuropsychology has recently transitioned to a more empirical approach to identify cognitive phenotypes associated with AD and TLE. Using data science methods, studies of AD have identified a number of phenotypes presenting with generalized cognitive deficits or focal profiles of impairment in memory, language, or other function. These phenotypes may differ in rates of progression and can be distinguished by unique genetic and biomarker profiles (67, 68). A similar literature in TLE has yielded a set of 3–4 cognitive phenotypes initially identified by Hermann and colleagues (55) and replicated, and differ in rates of cognitive decline and brain atrophy (69, 70).

Historically, study of neurocognitive impairment in TLE has focused on children and younger adults. Attention is shifting to older patients with longstanding epilepsy (EOE) and/or those with LOE to better understand how decades of seizures contribute to cognitive decline (71). Surprisingly, patients with EOE demonstrate a pattern of impairment on neuropsychological tests analogous to the decline seen in LOE to and aMCI patients (17). However, direct comparisons of MCI and TLE groups find that MCI patients exhibit greater impairment on tests of delayed memory while LOE patients have a more widespread profile of deficits in language, executive dysfunction, and visuospatial skills (18, 72, 73).

Several studies have demonstrated accelerated cognitive decline in patients with epilepsy, correlating with findings of increased atrophy on neuroimaging (2, 74). Research progress has been hindered over the years by a lack of an accepted taxonomy to classify cognitive disorders in patients with epilepsy across the lifespan (75). Studies using methods for diagnosing MCI in non-epileptic populations have found that approximately 60% of older individuals with epilepsy would meet diagnostic criteria for MCI (72, 73). Questions have arisen whether these findings are reflective of the effects of early cognitive deficits interacting with effects of normal aging, an accelerated form of aging, or chronic accumulation of environmental and health-related factors that reduce cognitive reserve (76).

While there is significant overlap in AD and TLE cognitive profiles, subtle differences exist. Patients with neurodegenerative conditions would be expected to decline over time while patients with well controlled epilepsy may not have significant memory decline. A more sophisticated understanding of their pathological, anatomical, and neurophysiological profiles could guide clinical phenotyping and diagnosis, especially at early stages of cognitive decline or seizure presentation.

## 4 Clinical diagnosis and differentiation

For guidance on diagnosing Alzheimer’s Disease and late-onset epilepsy, we refer readers to published guidelines (77, 78). However, even clinicians who diagnose and manage dementia or epilepsy may have difficulty recognizing seizures in AD patients or vice versa, especially at initial presentation. We offer several recommendations based on this review of the literature and our clinical experience with both populations.

1. *A careful medical and family history should be taken* to identify vascular causes which can cause cognitive decline or contribute to accelerated AD; sleep apnea; alcohol and drug use; family history of early onset dementia and traumatic brain injury (which causes both cognitive decline and seizures).
2. *Patients with amnestic MCI or early AD often have difficulty with both information encoding and retrieval, whereas patients with LOE primarily have impairments in retrieval, especially during delayed recall.* This observation is consistent with the concept of differential subfield vulnerability at early stages (29). This subtle difference can be assessed during the MMSE or MOCA during the clinical visit. During verbal encoding of 3 or 5 words, patients with aMCI or AD struggle to learn words, require multiple registration trials, and demonstrate difficulty with retrieval. LOE patients show more selective weakness in delayed retrieval. Neuropsychological evaluation should focus on whether memory impairment is isolated to difficulty with consolidation (more likely to be TLE only) or difficulty with all stages of memory function, including encoding and retrieval (more likely to be AD); other cognitive domains such as language and executive function are often more affected in neurodegenerative conditions than in isolated LOE.
3. *Clinical follow-up is important.* The degree and pace of cognitive decline is often faster in patients with aMCI or AD than in LOE patients (18). Patients with well controlled LOE are more likely to remain cognitively stable over time if their seizures and other medical issues are well controlled.
4. *When patients with aMCI and AD report a history of fluctuating mental status, or discrete episodes of altered awareness, agitation, or psychosis, seizures should be suspected*. Mesial temporal lobe onset seizures (mTLE) can present with psychic auras of anxiety and déjà vu, or viscero-sensory sensations with nausea, “butterflies,” and epigastric rising. Seizures can occur with or without alteration in awareness (focal impaired aware or focal unimpaired aware) and result in behavioral arrest. Seizures typically last from seconds to a minute, and rarely continue past 1–2 min. Seizures involving mTLE can progress to include obvious motor signs, such as posturing, repetitive clonic jerking (unilateral or bilateral, or tonic stiffening). Patients may be lethargic, confused, agitated, or even psychotic after seizures. Occult seizures should be suspected when MCI and AD patients become suddenly agitated or psychotic, or demonstrate fluctuating mental status (although Lewy Body Dementia could also be in the differential). Nocturnal seizures should be suspected when the patient wakes up confused or disoriented.
5. *We recommend an MRI Brain* for all patients who report memory or cognitive issues, and patients presenting with late-onset epilepsy. Besides obvious structural abnormalities, special attention should be paid to hippocampal volumes, white matter disease, lobar specific atrophy, or generalized atrophy that could point to underlying AD (bilateral hippocampal/temporal; parietal) or another neurodegenerative pathology. When the MRI Brain is structurally normal, and a neurodegenerative condition is strongly suspected, we recommend a PET MRI Brain to assess for lobar-specific dysfunction.
6. *We recommend ambulatory EEG (24 h) in all patients*, given that interictal epileptiform discharges (IEDs), especially from temporal lobe, are facilitated during non-REM Sleep (60, 79). Patients with temporal lobe epilepsy typically have unilateral spikes, whereas patients with aMCI or AD have bilateral hyperexcitability (16). AD patients with clinical seizures have characteristic IEDs seen on ambulatory EEG—discharges are bilateral, small and spiky in appearance, frequent, and occurring in wakefulness and REM sleep (16). Even with a normal ambulatory EEG, there may undetected interictal epileptiform discharges and silent seizures in AD patients that are only detected with invasive recordings (7). Besides IEDs, other EEG changes are apparent in aMCI and early AD. Bilateral frontotemporal slowing and mild slowing and desynchronization in the posterior dominant rhythm have been linked to the degree of amyloid and tau deposition, respectively (19). Sleep EEG in patients with temporal lobe epilepsy shows decreased spindle density (61). While mesial temporal IEDs can be difficult to detect with conventional scalp EEG, our center has had improved sensitivity with adding subtemporal (T1/T2) leads.

Taking the next step will require development of more sensitive and automated cognitive assays. Current neuropsychological tests are reliant on outdated models of cognitive functioning and depend on paper-pencil format (52, 80, 81). There is a clear need to “update testing” to reflect more contemporary cognitive models of development and administration through digital formats such as computers or smartphones, and could facilitate more frequent testing. Empirical study of cognitive phenotypes using network and artificial intelligence (AI) approaches can be used to efficiently and objectively analyze test findings (82, 83).

## 5 Novel directions in memory assessment

An ideal clinical assessment could (1) differentiate between early stages of AD and LOE, (2) detect early and subtle forms of memory impairment and precisely measure cognitive performance over time, and (3) be scaled to widespread patient populations. Testing would capture clinically meaningful memory behaviors, disambiguate language from memory, measure these behaviors sensitively and objectively, and allow serial assessment over time (84).

Subjective memory impairment, which correlates with initial amyloid accumulation in the brain, can precede AD dementia ascertainment by up to 18 years (56). Yet these subtle declines may not be detected by current neuropsychological testing methods. Many of the current cognitive assessments have been criticized as labor-intensive, subjective, and data-poor estimates of human behavior (52, 85, 86). In contrast to current practice of testing word lists, pairs, paragraphs, and abstract drawings, patients report difficulty with episodic memory, or remembrance of personally experienced events. Episodic memory under real-world circumstances binds perceptual details, spatial context, and temporal order to specific events (87), which may be missed in standard neuropsychological tests.

For example, development of tasks that measure subfield-specific functions could be useful for early diagnosis, phenotyping, and tracking. Given the differing cell types and differential functions of the hippocampal subfields, and differential patterns of decline in early AD, LO-TLE, and normal aging, some have proposed a functional map of the hippocampus, along the transverse (subfield-level) and longitudinal (anterior–posterior) axes (Figures 3, 4) (29). Combining these behavioral insights with automated segmentation of hippocampal subfields allows more precise functional-anatomical correlations (88). For example, CA3 is thought to be responsible for pattern integration (29), while dentate gyrus performs pattern separation (i.e., distinction between similar features or events). Some studies demonstrate decreased pattern separation in older individuals related to DG dysfunction with aging (30).

Future assessments could embrace more complex, naturalistic memory paradigms and computational analysis to make scoring more objective, sensitive, and quantifiable. Moreover, ideal testing could disambiguate language from memory function. Here, we highlight two promising directions in cognitive testing.

### 5.1 Eye tracking

Eye tracking, or the measurement of saccades, fixations, and pupillometry with high spatiotemporal precision, is an ideal method to readout brain-behavior relationships (57, 89). While rodents use mainly olfaction and locomotion to explore their environment, humans and other primates primarily depend on vision to extract and remember information about the world. Eye movements shape what is encoded – by chunking a continuous visual stream of information to deliver to widespread brain regions, including the hippocampus. Eye movement can track hippocampal activity at the millisecond time scale, as demonstrated by recent studies combining oculomotor measurements and hippocampal depth recordings in surgical epilepsy patients (90).

**Figure 5:**
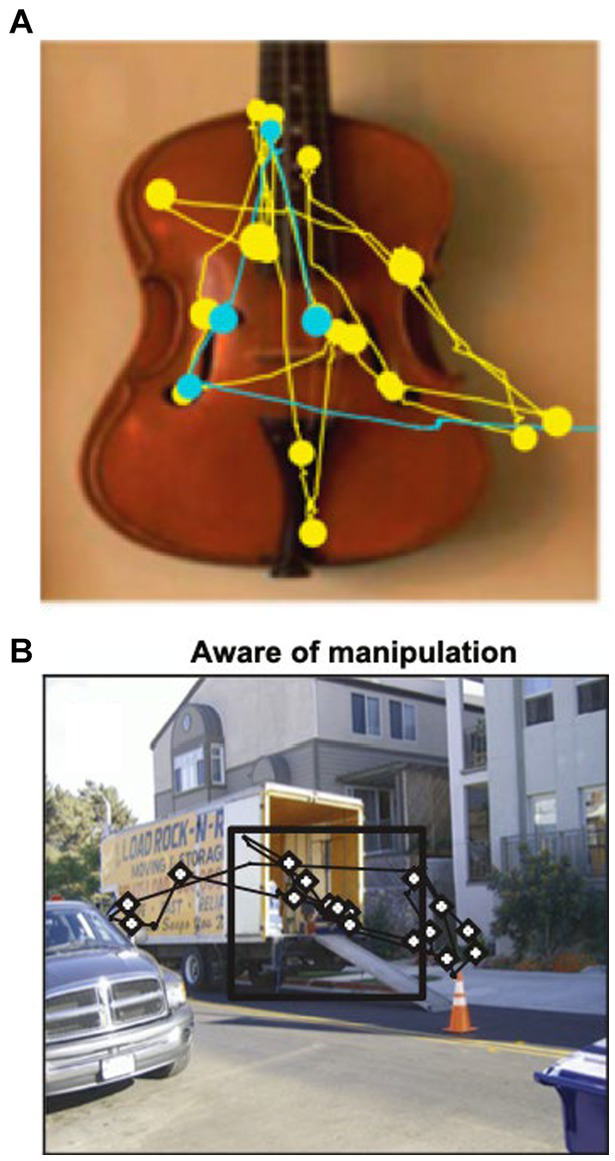
**(A)** Representative scan path showing that a macaque spends more time looking at the image during the first viewing (yellow) compared to second viewing (blue). Circles represent fixations; lines represent saccades. Adapted from Jutras et al. (89). **(B)** Macaques fixate more frequently on a manipulated (novel) scene area (inside black square). From Smith et al. (90).

When scanning the environment, eye movements rapidly switch between saccades and fixations. Saccades are sudden, ballistic eye movements between objects or features in the environment, while fixations are prolonged gaze on attended objects. Eye movements are not random but influenced by visual properties of the object (e.g., color, contrast) and past experience (i.e., episodic and semantic memories). For example, monkeys, human infants, and healthy adults prefer looking at novel vs. familiar objects (91–94). More gaze fixations occur within new vs. repeated viewing (Figure 5A) or within manipulated sections of the scene (Figure 5B), even if the subject is unaware of the manipulation (95–97). In contrast, patients with hippocampal damage have impaired novelty preference, manifest as equal time spent looking at new and old objects (98–100).

Besides novelty detection, eye movements reveal relational memory between objects (99, 101–103) and temporal sequences (104). Finally, eye tracking could provide a means to disambiguate language from memory testing. The strong preference for novelty manifested through gaze preference has been proposed to be a useful means of tracking memory changes in the preclinical and clinical AD populations (92, 105).

### 5.2 Spontaneous recall and natural language processing

While memory impairment is characteristic of AD presentation, patients may also present with subtle language decline. Word-finding difficulties and a restricted lexicon result in “empty speech” or verbose but incoherent speech (106–109).

Machine and deep learning methods applied to patient spontaneous speech have been applied to help in diagnosis of psychosis (107) and schizophrenia, and could be useful for AD diagnosis can aid in AD diagnosis (109–111). Several studies have leveraged publicly available speech samples. The DementiaBank corpus of speech samples was collected between 1983 and 1988 from healthy and cognitively impaired patients at the University of Pittsburgh (111). Clinical information including MMSE, neuropsychological and physical assessment, and clinical records were used to classify patients as possible or probable AD (167 participants). Control subjects (*n* = 167) were also included. The Cookie Theft picture description task from the Boston Diagnostic Aphasia Examination was used to elicit spontaneous speech, then transcribed at the word level, segmented into utterances, and annotated with pauses, paraphasias, and unintelligible words. Several of the studies using automatic, natural language processing-based features extracted from DementiaBank samples are summarized in Table 2.

**Table 2:** Examples of automated speech analysis in Alzheimer’s disease.

Publication	Patient groups	Main finding	Methods
Orimaye et al. (2017) (108)	Probable AD (*n* = 99)	Probable AD group had less use of syntactical components and greater use of lexical components in language compared to Healthy Controls (HCs). Less use of n-grams (combinations or sequences of words that create a unit of meaning) in probable AD group than in HCs.	DementiaBank language transcript clinical dataset (111). Automatic extraction of lexical, syntactic, and n-gram features of transcripts.
	Healthy Controls (*n* = 99)		
Yeung et al. (2021) (114)	Healthy controls (*n* = 10)	Greater severity in word-finding difficulty and incoherence in MCI and AD compared to controls. Automatically extracted features such as decreased word length and speech rate and increased pause frequency and length most strongly correlated with clinician ratings of WFD.	DementiaBank speech samples (111). 5 clinicians blindly rated each speech sample on word finding difficulty, incoherence, perseveration, and speech errors, on a Likert scale from zero (nL) to 3 (severe impairment). Automatic extraction of lexical, syntactic, semantic, and acoustic properties.
	MCI (*n* = 10)		
	AD (*n* = 10)		
Fraser et al. (JAD, 2016) (115)	Healthy controls (*n* = 97)	Built a model which discriminates between HCs and possible/probable AD with 81% accuracy. Semantic impairment, acoustic abnormality, syntactic impairment, and information impairment predict dementia diagnosis.	DementiaBank speech samples (111). Considered 370 features including syntactic complexity, grammar, vocabulary richness, lexical content, repetitiveness, and acoustic.
	Possible and Probable AD (*n* = 167)		
Beltrami (Front. Aging Neurosci 2018) (116)	Cognitively Impaired (*n* = 48: 32 MCI, 16 early dementia)	Acoustic features most altered in the patients compared to controls (including speech rate and pauses, and spectral properties). Lexical features differentiate early dementia patients (e.g., fewer content words and modifiers). Syntactic features (e.g., sentence complexity, fewer embedded phrases) decreased in early dementia and MCI patients.	Prospective study of spontaneous speech during description of a picture, typical working day, and last remembered dream. Automatic extraction of lexical, rhythmic, acoustic, and syntactic features of speech.
	Healthy Controls (*n* = 48)		

AD patients typically demonstrate slowed speech rate, word finding, and word retrieval difficulty (111, 112). One study using natural language processing (NLP) analyzed speech samples of 99 patients with probable AD to 99 healthy controls (108). Low-level features such as simpler syntactic structure (i.e., arrangement of words and phrases to create meaningful sentences) and decreased use of lexical components [i.e., autonomous units of language, such as words, prefixes (pre-, post-), suffixes (-s, -ing)] could differentiate AD patients from healthy controls (108). Another study found that linguistic features of descriptive speech (Cookie Theft task) in AD patients showed acoustic differences and semantic, syntactic, and informational differences, compared to healthy elderly (113). NLP methods applied to natural speech demonstrate that syntactic complexity combined with traditional neuropsychological test scores can differentiate between healthy elderly and MCI with high accuracy (>80%) (117).

To our knowledge, there have been no automated analyses of speech from epilepsy patients to detect cognitive changes. Given the widespread cognitive effects that have been discovered in patients with TLE (55), especially arising from the dominant lobe (62), word finding and speech changes would be expected. One study combined a questionnaire survey with NLP analysis of patients’ descriptions of their most recent description of transient loss of consciousness could predict a seizure or non-epileptic event with 85.5% accuracy (*n* = 21 epilepsy patients, *n* = 24 non-epileptic patients) (118).

Existing linguistic tools and insights into AD decline could be leveraged to quantify memory impairment. For example, the face-name task is an ecologically valid behavioral task that correlates with degree of amyloid burden in anterior hippocampus and limbic regions in healthy elderly individuals (119). However, to our knowledge, there are no existing tools to assess memory impairment using automated methods, that are both sensitive and scalable.

## 6 Conclusion

Results of MRI, pathological, neurophysiological, and behavioral studies demonstrate significant overlap between AD and LOE. Understanding the pathophysiological profiles of each disease can aid clinical detection at early disease stages, or once a primary diagnosis is made, recognize the presentation of a second diagnosis. We highlight the cognitive differences between early AD and LOE, but emphasize the need for new testing approaches, including those utilizing eye tracking and natural language processing, to measure subtle changes in memory at the preclinical or early clinical stages.

## Author contributions

AL: Conceptualization, Writing – original draft, Writing – review & editing. WB: Conceptualization, Writing – original draft, Writing – review & editing.
